# Prediction models for progression and outcomes in degenerative cervical myelopathy: a systematic review and critical appraisal

**DOI:** 10.3389/fneur.2026.1894454

**Published:** 2026-08-21

**Authors:** Renkun Zhao, Bo Xu, Xiaokuan Qin, Peng Zhang, Guoliang Ma, Zhizhuang Wang, Dongsheng Yuan, Penghui Li, Jiacheng Zheng, Yaling Gao, He Yin, Bowen Yang, Xin Chen

**Affiliations:** 1 Wangjing Hospital of China Academy of Chinese Medical Sciences, Beijing, China; 2 Hunan University of Chinese Medicine, Changsha, China; 3 Department of Knee Joint, Luoyang Orthopedic Hospital of Henan Province (Henan Orthopedic Hospital), Luoyang, China

**Keywords:** degenerative cervical myelopathy, prediction model, PROBAST, risk of bias, systematic review

## Abstract

**Objective:**

This systematic review evaluated the methodological quality, predictive performance, risk of bias, and clinical applicability of prediction models for disease progression and postoperative outcomes in degenerative cervical myelopathy (DCM), aiming to provide evidence for model optimization and clinical translation.

**Methods:**

PubMed, Embase, and Web of Science were searched from inception to May 2026. Original studies developing or validating DCM prediction models were included. Data extraction followed CHARMS, risk of bias was assessed using PROBAST, and reporting quality was evaluated with TRIPOD.

**Results:**

Twelve studies (prospective and retrospective cohorts from China, the US, Canada, and the Czech Republic) were included. Modeling approaches ranged from conventional regression to machine learning algorithms, with AUCs from 0.53 to 0.93. Major predictors included age, baseline JOA/mJOA, symptom duration, smoking, comorbidities, and radiomic features. PROBAST revealed low bias in participants, predictors, and outcomes for most studies, except Kadanka et al. (2017) which showed high outcome bias; however, all studies had high analysis-domain bias due to inadequate sample-event matching, inappropriate continuous variable handling, inconsistent missing data management, and insufficient calibration reporting. Only five studies reported both discrimination and calibration, and four completed both internal and external validation. TRIPOD compliance ranged from 59.1 to 81.8%, with sample size planning, calibration reporting, and model coefficients being most underreported. Clinical translation remains limited.

**Conclusion:**

Current DCM prediction models carry high overall bias and methodological heterogeneity. Parsimonious clinical models using readily available predictors (age, baseline mJOA, symptom duration) with rigorous validation offer advantages in calibration and interpretability over high-dimensional radiomic machine learning models. Future research should prioritize external validation of simple models and development of bedside-friendly tools, with adherence to TRIPOD and PROBAST standards.

**Systematic review registration:**

https://www.crd.york.ac.uk/PROSPERO/view/CRD420261407781, CRD420261407781.

## Introduction

Degenerative cervical myelopathy (DCM) represents the most common cause of non-traumatic spinal cord dysfunction in adults and ranks among the most severe degenerative cervical spine disorders (1). According to international spinal guidelines, DCM is predominantly characterized by progressive neurological deterioration resulting from spinal cord compression, accompanied by gait disturbances, impaired fine motor function of the hands, and reduced quality of life (2). Global systematic reviews have demonstrated that the prevalence of DCM among middle-aged and elderly populations rises year by year. Given its high risk of disability, DCM has become a major spinal disorder threatening public health worldwide. As it arises from chronic biomechanical overload and mechanical compression of the cervical spinal cord, representing a core research object of translational spine biomechanics and clinical engineering (3).

The natural history of DCM and perioperative recovery constitute two core components of its clinical management, and multiple factors can influence disease progression and surgical outcomes (4). Accumulating evidence has indicated that the rate of DCM progression and postoperative prognosis are closely associated with a wide range of risk factors, including age, baseline neurological function, severity of spinal cord compression on imaging, intramedullary signal changes, and comorbidities (5, 26). Failure to identify patients in a timely manner at high risk of rapid disease progression or poor prognosis may lead to missed optimal intervention windows, thereby causing irreversible neurological deficits. This not only severely impairs the quality of life of patients and their families but also imposes a substantial medical and socioeconomic burden (6). Accordingly, early and accurate identification of DCM patients with a high risk of disease progression and adverse prognosis is critical for optimizing intervention timing and improving clinical outcomes.

Clinical prediction models have been widely adopted in spinal surgery in recent years. Based on patients’ baseline characteristics, clinicians can quantify the risks of disease progression and unfavorable postoperative outcomes, which may offer references to support for individualized clinical decision-making. Such models help improve medical efficiency and the accuracy of prognostic assessment, and further guide the selection of intervention timing and surgical strategies (7–9). Developing multi-variable prediction models for DCM progression and prognosis can clarify the synergistic effects of multiple influencing factors, facilitate evidence-based understanding of the natural history and recovery process of DCM, support the formulation of stratified management strategies, and may help lower the risk of neurological deterioration and poor prognosis.

Prediction models for DCM progression and prognosis enable clinicians to screen high-risk patients, and implement stratified monitoring, early intervention and individualized surgical planning in a timely manner, so as to effectively slow disease progression and improve postoperative functional recovery (10). A number of studies at home and abroad have developed and validated DCM-related prediction models for diverse populations. Nevertheless, the methodological quality and evidence strength of these existing models remain unclear (11). Prior systematic reviews focusing on spinal prediction models have consistently reported pervasive methodological limitations and high overall bias risk, yet none have comprehensively compared the practical value of high-dimensional radiomic signatures against readily available clinical predictors, ranked modeling approaches based on combined calibration and external validation performance rather than isolated AUC values, or established clear stratified research priorities for clinical and computational investigators. Therefore, the present study systematically summarizes and evaluates published studies on prediction models for DCM progression and prognosis, aiming to provide references for the optimization, external validation and clinical translation of relevant prediction models in future research, with a particular emphasis on generating integrated, actionable translational recommendations to address the above unmet research demands.

## Methods

Literature screening and data extraction were performed in accordance with the Checklist for Critical Appraisal and Data Extraction for Systematic Reviews of Prediction Modeling Studies (CHARMS) (12). The Prediction Model Risk of Bias Assessment Tool (PROBAST) was adopted to evaluate the risk of bias and applicability of primary studies on prediction models for the progression and prognosis of DCM (13).

The reporting standard for Transparent Reporting of a multivariable prediction model for Individual Prognosis Or Diagnosis (TRIPOD) was used to assess the methodological standardization of DCM prediction model development (14). Two independent researchers performed blind item-wise evaluation against the full 22-item TRIPOD checklist for each eligible article. Each checklist item was categorized as fully reported (FR), partially reported (PR), or not reported (NR). We calculated the overall reporting compliance rate for each study, defined as the proportion of fully satisfied TRIPOD items among all 22 criteria.

This systematic review was prospectively registered on PROSPERO (registration ID: CRD420261407781, available at https://www.crd.york.ac.uk/PROSPERO/view/CRD420261407781).

### Search strategy

A comprehensive electronic literature search was conducted across PubMed, Embase and Web of Science, covering the period from database inception to May 1, 2026.

The search strategy combined Medical Subject Headings (MeSH) with free terms in titles, abstracts and keywords. Search terms focused on the study population (DCM) and research themes, including disease progression, clinical prognosis, risk prediction, prediction model, nomogram, risk score and machine learning. The whole review workflow strictly adheres to the PRISMA 2020 reporting guideline for systematic reviews. Full electronic search strategies of PubMed, Embase and Web of Science are provided in Supplementary File S1 for transparent replication.

### Literature screening and eligibility criteria

Duplicate records identified from database searches were removed using EndNote software. Two independent investigators initially screened the titles and abstracts of remaining articles. Full-text evaluation was subsequently conducted independently by the two researchers to determine whether studies met the inclusion criteria.

Inclusion criteria: (1) participants were patients with a confirmed diagnosis of DCM; (2) studies developed or validated prediction models for DCM progression or clinical outcomes including postoperative functional recovery, complications and reoperation, with complete procedures for model construction, validation and evaluation; (3) original clinical studies focusing on the development or validation of prediction models; (4) articles published in English.

Exclusion criteria: (1) studies only exploring influencing factors of DCM without constructing prediction models; (2) reviews, conference abstracts, systematic reviews, meta-analyses, letters, case reports and expert opinions; (3) basic cell or animal experiments, pure imaging analyses, and univariate analyses without model development and validation; (4) studies enrolling non-DCM populations or patients complicated with spinal cord tumors, trauma or inflammatory myelopathy; (5) duplicate publications, low-quality studies and articles with unavailable raw data.

Any discrepancies between the two investigators were resolved through consultation with a third researcher to reach a consensus. Cross-check of all included studies was also performed to guarantee the accuracy of screening results.

### Data extraction

Data extraction was completed independently by two researchers and cross-checked by a third investigator. Disagreements were resolved via group discussion or consultation with professional experts. A standardized data extraction form was established based on CHARMS, and the following information was extracted: (1) basic characteristics of included studies: publication year, country, study design, participants, research setting and follow-up duration; (2) predicted outcomes: definitions and diagnostic criteria for DCM progression, postoperative neurological recovery, complications, reoperation and other endpoints; (3) details of model development: number of candidate predictors, processing methods for continuous variables, sample size, number of outcome events, volume and management of missing data, model construction approaches and variable selection strategies; (4) model performance and predictive factors: methods for performance assessment and validation, main predictive factors, model presentation forms, as well as strengths and limitations of each study.

Model performance was evaluated in terms of discrimination and calibration. The area under the receiver operating characteristic curve (AUC) was used to assess discrimination. An AUC value of 0.5 indicated no predictive ability, while a value of 1 represented perfect predictive performance. Calibration was evaluated using calibration curves or the Hosmer–Lemeshow goodness-of-fit test. In the calibration curve, the X-axis represented predicted probabilities and the Y-axis represented observed probabilities. A satisfactorily calibrated model usually presents an intercept near 0 and a slope close to 1. The Hosmer–Lemeshow test was applied to examine the statistical difference between predicted and actual event probabilities.

Validation is an indispensable step in prediction model development, which is divided into internal validation and external validation. Internal validation was commonly implemented via data splitting or bootstrap resampling. External validation was used to evaluate model generalizability, including temporal validation, geographical validation and domain validation.

Notably, substantial heterogeneity existed in the primary predictive targets across eligible studies. We classified all models into two distinct subgroups according to their defined endpoints: (1) disease progression models: Predict conversion from asymptomatic cervical cord compression to symptomatic DCM; (2) postoperative prognosis models: Predict surgical outcomes including mJOA/JOA recovery, neck VAS pain relief and MCID attainment. Subgroup comparisons of model performance and methodological quality were performed based on this stratification to avoid overgeneralized pooled conclusions across heterogeneous endpoints.

### Assessment of risk of bias and applicability

Risk of bias assessment is a key component of this systematic review. Two researchers independently evaluated the risk of bias and applicability of included DCM prediction model studies using the PROBAST tool. Designed specifically for systematic reviews on prognostic and diagnostic multivariable prediction models, PROBAST consists of four core domains: participants, predictors, outcomes and analysis. Risk of bias was assessed across all four domains, whereas applicability was judged based on the first three domains.

Twenty signalling questions were used to comprehensively assess the risk of bias. Each question was answered as Yes (Y), Probably Yes (PY), Probably No (PN), No (N) or Insufficient Information (NI). Each domain and the overall study were rated as low risk of bias, high risk of bias, or unclear risk of bias. Applicability was classified as good, poor or unclear.

Overall judgment was defined as follows: studies with low risk of bias and good applicability across all domains were rated as having low overall risk of bias and good applicability. Studies presenting high risk of bias or poor applicability in any single domain were regarded as having high overall risk of bias or poor applicability. If any domain was rated as unclear while the remaining domains were low risk or good applicability, the overall assessment was defined as unclear risk of bias or unclear applicability. Prior to assessment, the two investigators received standardized training and conducted group discussions to ensure consistent understanding and application of the PROBAST tool. Any scoring discrepancies arising during independent PROBAST evaluation were resolved via consultation with a third senior reviewer, adopting the identical arbitration process used for literature screening and data extraction.

## Results

### Literature screening

**Figure 1:**
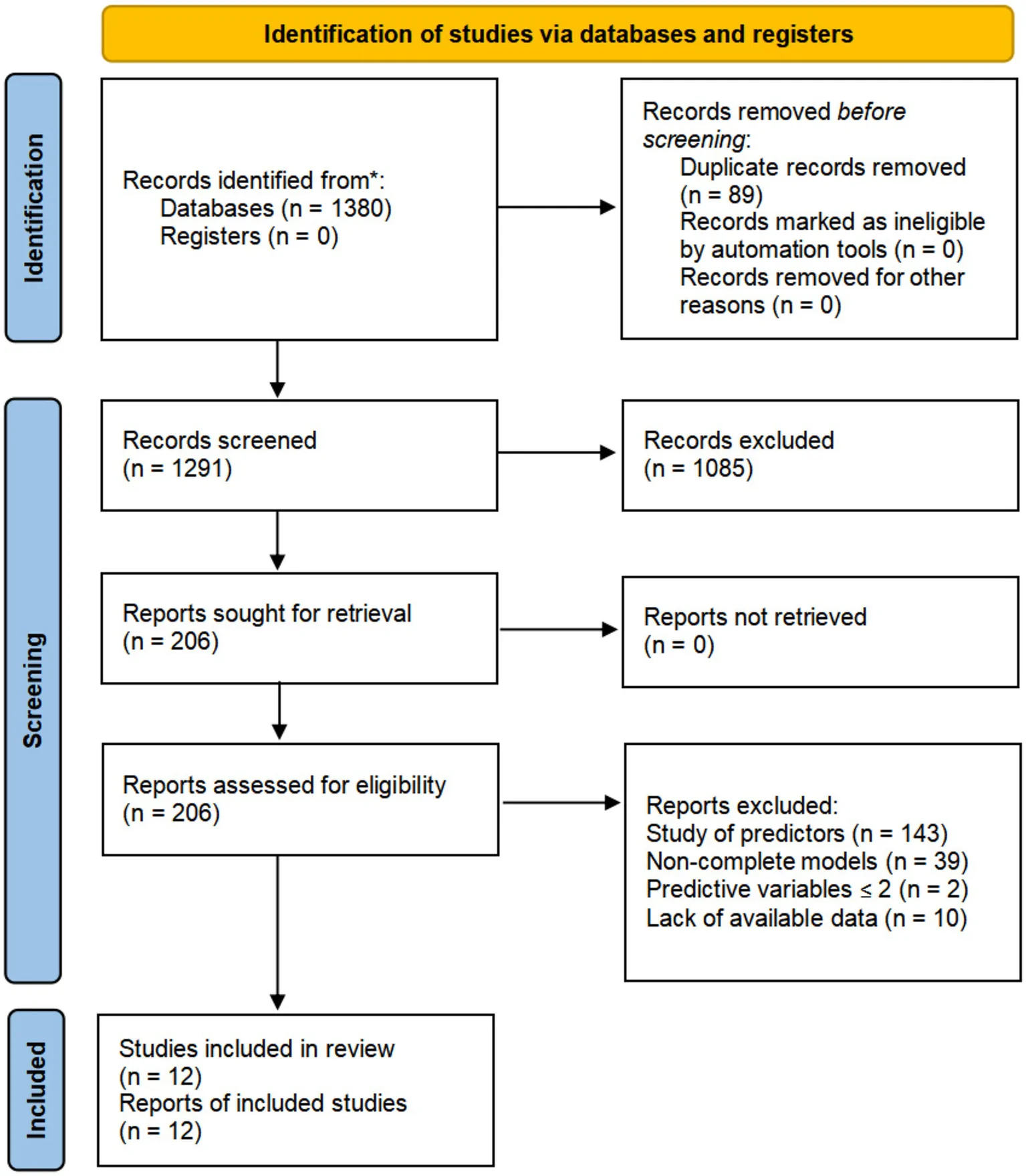
Flowchart of the study selection process.

A total of 1,380 articles relevant to DCM prediction models were retrieved through comprehensive searches. After stepwise screening, 12 studies were finally included. Firstly, 89 duplicate records were removed. By screening titles and abstracts, 781 articles with ineligible study types and 304 articles with irrelevant research topics were excluded. The remaining 206 articles were assessed via full-text reading. Among them, 143 studies merely explored influencing factors without constructing prediction models, 39 had incomplete procedures for model development and validation, two studies included fewer than two predictive variables, and 10 had unavailable raw data, all of which were excluded. Ultimately, 12 studies (5, 10, 15–24) that fully met the inclusion criteria were enrolled in this systematic review. The literature screening flowchart is presented in Figure 1.

### Basic characteristics of included studies

Twelve studies were included in the present analysis, eight (10, 16, 18, 20–24) of which were published within the past 5 years. These studies were conducted in four countries: China, the United States, Canada and the Czech Republic. In terms of study design, seven (5, 15–17, 19, 20, 22) were prospective cohort studies and five (10, 18, 21, 23, 24) were retrospective cohort studies. Regarding study populations, one study (17) focused on disease progression risk in patients with degenerative cervical spinal cord compression without myelopathy, while the remaining studies enrolled patients with DCM who underwent cervical decompression surgery.

Data sources comprised affiliated university hospitals, tertiary hospitals, international multicenter prospective spinal cohorts and national clinical registration databases, covering both single-center and multicenter investigations. Clinical medical records and specialized spinal databases served as the primary data sources. All studies conducted follow-up assessments of postoperative neurological outcomes, with follow-up durations ranging from 3 months to 3 years, covering short-term (3–6 months), medium-term (1–2 years) and long-term (3 years) observation periods.

Postoperative outcomes were evaluated using multiple heterogeneous indicators, including the Japanese Orthopaedic Association (JOA) score, modified Japanese Orthopaedic Association (mJOA) score, stratification of postoperative neurological function, visual analogue scale (VAS) neck pain score, and achievement of the minimal clinically important difference (MCID). Notably, substantial heterogeneity existed in primary prediction targets: only Kadanka et al. (17) constructed a model to predict progression from asymptomatic spinal cord compression to symptomatic DCM, while the other 11 studies (5, 10, 15, 16, 18–24) all built prognostic models for postoperative recovery after cervical decompression surgery. Detailed endpoint definitions for each study are summarized in Table 1.

**Table 1:** Basic characteristics of the included studies.

First author (year)	Country	Study design	Target population	Data Sources	Follow-up time	Outcome measure
Zhou (2025) (10)	China	Retrospective cohort study	Patients with DCM undergoing cervical decompression surgery	Peking University Third Hospital	3–6 months postoperatively	Achievement of MCID in JOA score
Kadanka (2017) (17)	Czech Republic	Prospective cohort study	NMDCC patients	Single university hospital	Median 3 years	Progression to symptomatic DCM
Jaja (2023) (16)	Canada	Prospective cohort study	Symptomatic DCM patients receiving surgical decompression	International multicenter prospective cohorts	6, 12 and 24 months postoperatively	mJOA and SF-36 PCS postoperative recovery trajectories
Zhang (2022) (24)	China	Retrospective cohort study	DCM surgical patients	Single tertiary hospital	3 years postoperatively	Postoperative neurological recovery rate stratification
Merali (2019) (19)	Canada	Prospective cohort study	Symptomatic DCM patients undergoing surgical decompression	AO Spine multicenter prospective cohorts	6, 12 and 24 months postoperatively	MCID achievement of SF-6D and mJOA scores
Khan (2021) (18)	Canada	Retrospective cohort study	Symptomatic DCM patients undergoing cervical decompression	AO Spine multicenter databases	1 year postoperatively	Postoperative decline in mJOA functional status
Song (2024) (21)	China	Retrospective cohort study	Symptomatic DCM patients undergoing surgical decompression	Three independent medical centers	1 year postoperatively	Postoperative JOA recovery rate classification
Stephens (2023) (22)	USA	Prospective cohort study	DCM patients undergoing elective cervical surgery	National clinical registry database	1 year postoperatively	Postoperative mJOA sub-domain improvement
Zhang (2021) (23)	China	Retrospective cohort study	DCM surgical patients	Single tertiary hospital	3 years postoperatively	Postoperative mJOA dichotomous prognosis
Tetreault (2015) (5)	Canada	Prospective cohort study	Symptomatic DCM patients undergoing surgical decompression	AO Spine multicenter cohorts	1 year postoperatively	Achievement of optimal mJOA (≥16 points)
Evaniew (2020) (15)	Canada	Prospective cohort study	Symptomatic DCM surgical patients	Canadian Spine Research Network	1 year postoperatively	MCID achievement of mJOA score
Saggi (2025) (20)	USA	Prospective cohort study	DCM patients undergoing elective cervical surgery	Quality Outcomes Database	2 years postoperatively	MCID achievement of neck pain VAS score

DCM, degenerative cervical myelopathy; MCID, minimal clinically important difference; JOA, Japanese Orthopaedic Association Score; mJOA, modified Japanese Orthopaedic Association Score; SF-36 PCS, 36-Item Short Form Health Survey Physical Component Summary; SF-6D, 6-Dimension Short Form Health Survey; VAS, visual analogue scale; AO Spine, AO Foundation Spine Division; NMDCC, Neurological Manifestations of Degenerative Cervical Myelopathy Cohort.

The number of candidate predictive variables ranged from 7 to 1,032 across the 12 studies. Eight studies (10, 15, 18–21, 23, 24) retained the original values of continuous variables including age, disease duration, preoperative JOA/mJOA scores and body mass index (BMI), without categorization or transformation. The other four studies discretized continuous variables such as age, symptom duration and spinal cord compression severity using stratification, quartile grouping, restricted cubic splines or clinical cut-off values.

According to the missing data handling column in Table 2, two studies (16, 17) did not report any strategies for managing missing data (NR). The remaining 10 studies all explicitly reported their specific handling approaches, which can be broadly classified into two categories. Five studies (15, 20, 21, 23, 24) applied complete case analysis by directly excluding samples with missing data. The other five studies adopted various imputation or correction methods: Merali et al. (19) first removed predictors with missingness over 5% and then adopted the k-nearest neighbor algorithm to impute residual missing values; Khan et al. (18) simply used the k-nearest neighbor algorithm for data imputation; Tetreault et al. (5) used multiple imputation; Stephens et al. (22) corrected missing covariates via canonical variate regression; and Zhou et al. (10) assigned a negative value to all missing binary variables.

**Table 2:** Basic characteristics for constructing prediction models of DCM.

First author (year)	Candidate variables		Total number of participants		Missing data		Modeling methods	Method of variable selection
	Number	Methods for dealing with continuous variables	Number	Number of outcome events	Number	Processing method		
Zhou (2025) (10)	63	Continuous variables were left unaltered.	672	369	124	Missing binary variables were imputed as negative values.	LR, SVM, RF, XGBoost, LightGBM	SHAP
Kadanka (2017) (17)	20	Continuous variables were dichotomized using optimal cut-off values.	112	15	NR	NR	LR, Cox regression, Multivariate LR	FS
Jaja (2023) (16)	10	Age was divided into quartiles; other continuous variables remained unaltered.	757	382	93	NR	Logistic regression, Group-based trajectory modeling	LASSO
Zhang (2022) (24)	1,032	Continuous variables were left unaltered.	151	82	NR	Analysis with complete data.	ET	PCA + CE
Merali (2019) (19)	111	Continuous variables were centralized and scaled without discretization.	757	376	118	Variables with missing >5% were deleted; KNN imputation.	LR, SVM, RF, DT, ANN	RFE
Khan (2021) (18)	51	Continuous variables were centralized and scaled.	702	85	76	Missing values were imputed via KNN algorithm.	LR, SVM, GBM, PLS	RFE
Song (2024) (21)	11	Continuous variables were left unaltered.	406	370	36	Patients with missing data were excluded.	LR, GLM, LDA, PLS-DA, SVM, KNN, RF, DT, XGBoost	LASSO + ES
Stephens (2023) (22)	20	Continuous variables were fitted with restricted cubic splines.	8,470	5,000	3,470	Missing covariates were imputed with canonical variates.	Ordinal proportional odds regression	BS
Zhang (2021) (23)	1,032	Continuous variables were standardized with Z-score.	151	80	NR	Analysis with complete data.	SVM, RF, ET, GBDT, LDA, QDA, KNN	PCA, FA, ICA
Tetreault (2015) (5)	7	Continuous variables were categorized by clinical cut-off.	757	608	149	Missing data were imputed via multiple imputation.	LR	Stepwise
Evaniew (2020) (15)	10	Continuous variables remained unprocessed.	285	205	80	Patients with incomplete data were excluded.	Binomial LR	FW + BW
Saggi (2025) (20)	20	Continuous variables were left unaltered.	1,141	NR	NR	Analysis with complete data.	RF	RFE

LR, logistic regression; SVM, support vector machine; RF, random forest; XGBoost, extreme gradient boosting; LightGBM, light gradient boosting machine; DT, decision tree; ANN, artificial neural network; GBM, gradient boosting machine; PLS, Partial Least Squares; GLM, Generalized Linear Model; LDA, Linear Discriminant Analysis; PLS-DA, Partial Least Squares Discriminant Analysis; KNN, K-Nearest Neighbors; ET, extra trees; GBDT, Gradient Boosting Decision Tree; QDA, Quadratic Discriminant Analysis; SHAP, SHapley Additive exPlanations; FS, Forward Selection; LASSO, least absolute shrinkage and selection operator; PCA, principal component analysis; CE, conditional expectation; RFE, recursive feature elimination; ES, early stopping; BS, Backward Selection; FA, factor analysis; ICA, independent component analysis; FW, Forward Selection; BW, Backward Selection; NR, not reported.

### Model development and performance

Table 2 summarizes the general information on the development of the 12 prognostic prediction models for DCM. Five studies (5, 15, 20, 22, 24) adopted a single statistical method for model construction, while the other seven studies established combined models using 2 to 9 distinct methodologies.

The modeling approaches utilized in the included studies mainly included logistic regression, ordinal proportional odds regression, Cox regression, regularized logistic regression, naive Bayes, decision tree, random forest, gradient boosting, support vector machine, artificial neural network, extreme gradient boosting (XGBoost), light gradient boosting machine and extra trees. Given the diverse modeling strategies, the predictive variables incorporated in different models also differed greatly.

Common variable selection methods comprised direct variable inclusion, stepwise regression, pruning algorithm, filter-based feature selection, recursive feature elimination, least absolute shrinkage and selection operator (LASSO) regression, principal component analysis, canonical correlation analysis, factor analysis, independent component analysis, optimal subset selection, forward/backward stepwise selection, exhaustive search and SHapley Additive exPlanations (SHAP)-based feature screening.

In terms of model validation, validation strategies varied considerably across the 12 included studies. Four studies (5, 10, 23, 24) performed both internal validation (via data splitting, cross-validation, or bootstrap resampling) and independent external validation. Among the remaining studies, three (18–20) adopted only internal validation through random data splitting or k-fold cross-validation, and another three (16, 21, 22) conducted internal validation solely via bootstrap resampling. Notably, two studies (15, 17) did not report any formal internal or external validation procedures, as they only presented univariate or multivariate regression analyses without validation metrics.

The AUC values of predictive models ranged widely from 0.53 to 0.93, with clear discrepancy driven by heterogeneous clinical endpoints. The single model predicting preclinical DCM progression yielded an AUC range of 0.60–0.76 (17). Among the 11 postoperative prognostic models focused on mJOA/JOA, VAS and MCID outcomes, AUC values varied from 0.53 to 0.93, indicating highly variable discriminative performance across distinct postoperative clinical targets. For model calibration assessment, five studies (5, 16, 18, 21, 22) reported calibration assessments, including calibration plots, calibration curves and Hosmer–Lemeshow test.

Due to discrepancies in enrolled variables, modeling strategies and predictive performance across studies, the final identified prognostic predictors for DCM were not consistent. Some models adopted relatively complex structures. For instance, Zhang et al. (23) applied multiple dimension reduction and feature selection techniques including principal component analysis, factor analysis and independent component analysis to screen dozens of variables from hundreds of radiomic features for model establishment.

Overall, the main prognostic predictors for DCM included preoperative neurological function (JOA/mJOA scores, each domain of the 36-item Short-Form Health Survey [SF-36]), demographic factors (age, gender, BMI), disease-related indicators (disease duration, neck pain, smoking status, comorbidities), imaging and radiomic parameters (spinal cord cross-sectional area, spinal cord compression rate, T2*-weighted radiomic features), as well as surgery-related factors (posterior surgical approach, surgical procedures).

The prediction models were presented in various forms, such as conventional regression models, machine learning models combined with SHAP analysis, simplified risk scoring systems, radiomics-based prediction models, ordinal proportional odds regression models and random forest-based prediction calculators. All 12 studies elaborated the strengths and limitations of their respective models in detail. The performance metrics and predictive factors of all models are listed in Table 3.

**Table 3:** Performance and predictors of risk prediction models for DCM.

First author	Model performance		Model evaluation	Main predictors	Model presentation	Strengths and limitations
	AUC	Calibration method				
Zhou (10)	0.70–0.79	NR	3-fold cross-validation and external validation	Preoperative JOA, age, SF-36-PF, SF-36-BP, SF-36-GH	Machine learning model with SHAP analysis	Multiple machine learning models were compared and validated. The feature-reduced model was externally validated. However, the follow-up duration was short, and only clinical scale variables were included.
Kadanka (17)	0.60–0.76	NR	Univariate and multivariate regression analysis	Radiculopathy, spinal cord cross-sectional area, compression ratio	Regression prediction model	A prospective follow-up design with clear imaging cut-off values was used. However, the sample size was small and no formal model validation was performed.
Jaja (16)	0.62–0.77	Calibration plot	Bootstrap internal validation, LASSO feature selection	Age, neck pain, smoking status, posterior surgical approach	Simple risk scoring system	A large multicenter prospective cohort was adopted and trajectory analysis was applied. Good model calibration was demonstrated. However, external validation was lacking.
Zhang (24)	0.71–0.81	NR	Internal and external validation	Radiomic features, age, preoperative mJOA, symptom duration	Radiomics-based machine learning model	Radiomic predictors showed better predictive efficacy than conventional MRI indicators. However, single-center design limited generalizability.
Merali (19)	0.67–0.73	NR	10-fold cross-validation, RFE feature selection	Age, body weight, smoking status, preoperative mJOA, symptom duration	Machine learning predictive model	Large multicenter prospective data was adopted with multiple ML algorithms compared. However, imaging variables were not incorporated.
Khan (18)	0.68–0.83	Calibration curve	10-fold cross-validation, RFE feature selection	Preoperative mJOA, age, symptom duration, male gender, comorbidities	Machine learning prediction model	Multiple machine learning algorithms were compared with good model calibration. However, external validation was not performed.
Song (21)	0.73–0.93	Calibration plot	Bootstrap internal validation, LASSO and exhaustive selection	Age, sex, disease duration, preoperative JOA, imaging and inflammatory indicators	Multimodel machine learning model	Various statistical and ML models were comprehensively optimized. However, the study was retrospective with insufficient external verification.
Stephens (22)	0.74	Calibration plot	Bootstrap internal validation	Baseline mJOA, age, race, symptom duration, anxiety, smoking	Ordinal proportional odds regression model	A large national clinical registry was used with rigorous ordinal regression. However, high follow-up loss and no external validation existed.
Zhang (23)	0.53–0.74	NR	5-fold cross-validation and external validation	Age, preoperative mJOA, symptom duration, T2* radiomic features	Radiomics-based machine learning model	Multiple algorithms and feature reduction methods were screened. However, single-center sample limited extrapolation.
Tetreault (5)	0.74–0.77	Hosmer-Lemeshow test, Calibration plot	Bootstrap internal and external validation	Baseline mJOA, age, symptom duration, gait impairment, psychiatric comorbidities, smoking	Logistic regression clinical model	Multinational prospective cohorts achieved rigorous internal and external validation. However, psychiatric comorbidities had regional underreporting bias.
Evaniew (15)	NR	NR	Univariate and multivariate logistic regression	Age, baseline mJOA, symptom duration, smoking status	Clinical regression prediction model	Multicenter data was used for external verification of prognostic factors. However, AUC and calibration indicators were not reported.
Saggi (20)	0.80	NR	5-fold cross-validation	Age, baseline neck VAS, baseline mJOA, EQ-VAS, BMI	Random forest prediction calculator	Prospective database and recursive feature selection were adopted. However, external validation and imaging indicators were lacking.

DCM, degenerative cervical myelopathy; JOA, Japanese Orthopaedic Association Score; mJOA, modified Japanese Orthopaedic Association Score; SF-36-PF, 36-Item Short Form Health Survey Physical Function Subscale; SF-36-BP, 36-Item Short Form Health Survey Bodily Pain Subscale; SF-36-GH, 36-Item Short Form Health Survey General Health Subscale; VAS, visual analogue scale; EQ-VAS, EuroQol Visual Analogue Scale; T2*, T2-star Magnetic Resonance Imaging Sequence; AUC, area under the receiver operating characteristic curve; SHAP, SHapley Additive exPlanations; RFE, recursive feature elimination; LASSO, least absolute shrinkage and selection operator; ML, machine learning; MRI, Magnetic Resonance Imaging; BMI, body mass index; NR, not reported.

### Assessment of risk of bias and applicability

**Figure 2:**
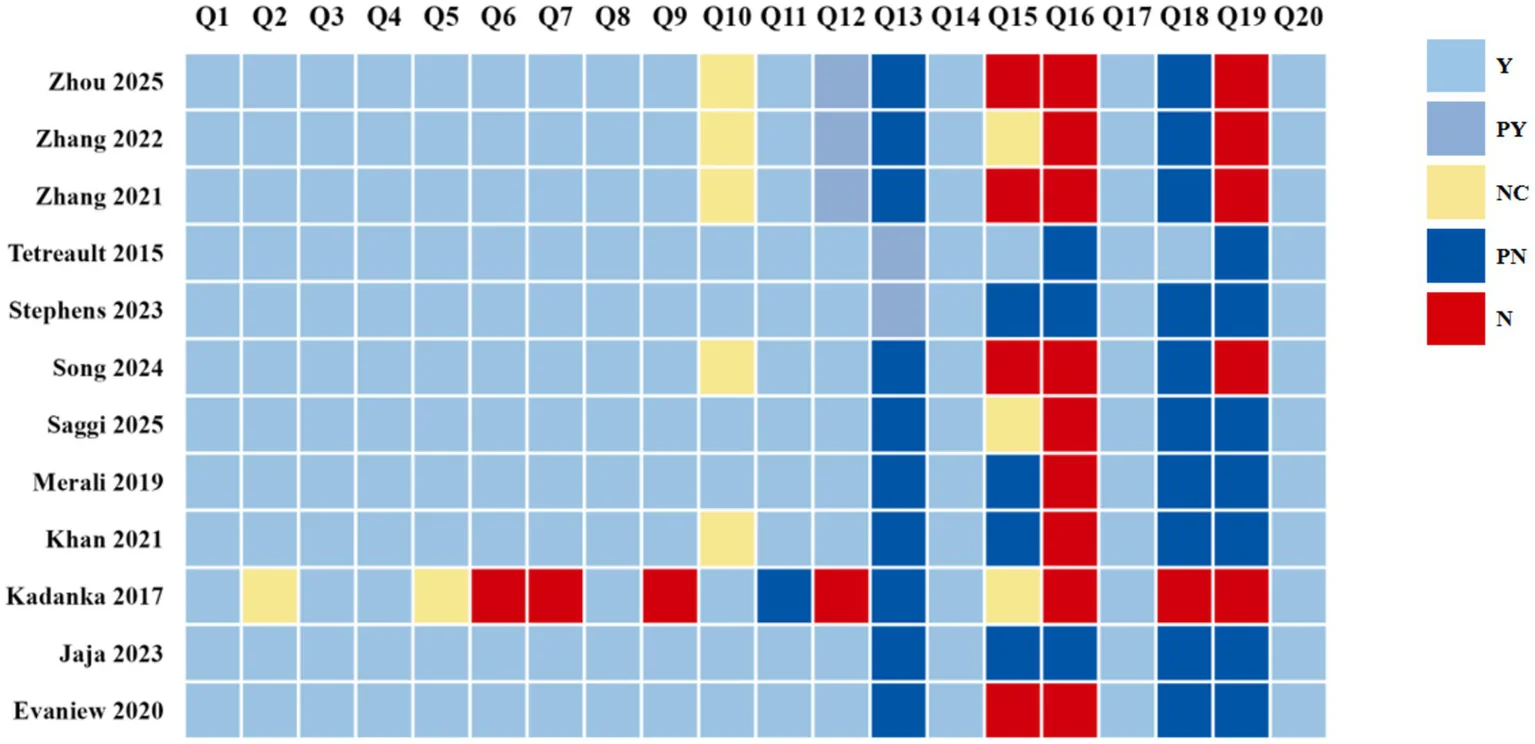
Risk of bias assessment (using PROBAST) based on four domains across 12 studies. Y, Yes; PY, Probably Yes; NC, not clear; PN, Probably No; N, No. Q1: Were appropriate data sources used, e.g., cohort, RCT, or nested case–control study data? Q2: Were all inclusions and exclusions of participants appropriate? Q3: Were predictors defined and assessed in a similar way for all participants? Q4: Were predictor assessments made without knowledge of outcome data? Q5: Are all predictors available at the time the model is intended to be used? Q6: Was the outcome determined appropriately? Q7: Was a prespecified or standard outcome definition used? Q8: Were predictors excluded from the outcome definition? Q9: Was the outcome defined and determined in a similar way for all participants? Q10: Was the outcome determined without knowledge of predictor information? Q11: Was the time interval between predictor assessment and outcome determination appropriate? Q12: Were there a reasonable number of participants with the outcome? Q13: Were continuous and categorical predictors handled appropriately? Q14: Were all enrolled participants included in the analysis? Q15: Were participants with missing data handled appropriately? Q16: Was the selection of predictors based on univariable analysis avoided? Q17: Were complexities in the data (e.g., censoring, competing risks, sampling of control participants) counted for appropriately? Q18: Were relevant model performance measures evaluated appropriately? Q19: Were model overfitting, underfitting, and optimism in model performance accounted for? Q20: Do predictors and their assigned weights in the final model correspond to the results from the reported multivariable analysis.

The results of risk of bias and applicability assessment based on the PROBAST tool are presented in Tables 4, 5, Figure 2. Collectively, all 12 studies were rated as having an overall high risk of bias, whereas they exhibited satisfactory performance regarding individual domains and general clinical applicability.

**Table 4:** Assessment of the risk of bias in participants, predictors, analysis, outcome domains, and overall.

First author	Risk of bias				Overall
	Participants	Predictors	Outcome	Analysis	
Zhou (10)	NC	L	L	H	H
Kadanka (17)	L	L	H	H	H
Jaja (16)	L	L	L	H	H
Zhang (24)	NC	L	L	H	H
Merali (19)	L	L	L	H	H
Khan (18)	NC	L	L	H	H
Song (21)	NC	L	L	H	H
Stephens (22)	L	L	L	H	H
Zhang (23)	NC	L	L	H	H
Tetreault (5)	L	L	L	H	H
Evaniew (15)	L	L	L	H	H
Saggi (20)	L	L	L	H	H

L, low risk of bias; H, high risk of bias; NC, not clear.

**Table 5:** Assessment of the applicability of the study participants, predictors, and outcome domains.

First author	Participants	Predictors	Outcome	Overall applicability
Zhou (10)	L	L	L	L
Kadanka (17)	NC	L	H	NC
Jaja (16)	L	L	L	L
Zhang (24)	L	L	L	L
Merali (19)	L	L	L	L
Khan (18)	L	L	L	L
Song (21)	L	L	L	L
Stephens (22)	L	L	L	L
Zhang (23)	L	L	L	L
Tetreault (5)	L	L	L	L
Evaniew (15)	L	L	L	L
Saggi (20)	L	L	L	L

L, low risk of bias; H, high risk of bias; NC, not clear risk of bias.

In the participants domain, seven studies (5, 15–17, 19, 20, 22)—all of which adopted prospective cohort designs with explicit enrollment criteria and clear diagnostic definitions for DCM—were rated as low risk of bias. However, the remaining five retrospective cohort studies (10, 18, 21, 23, 24) were rated as unclear risk in this domain. Although these retrospective studies also documented enrollment standards, they derived data from non-consecutive, single-center medical records, and most did not provide sufficient detail regarding the sequence of participant selection or whether enrollment was consecutive. Such omissions introduce potential selection bias and unmeasured confounding, which cannot be fully ruled out without transparent reporting.

In the predictors domain, all 12 studies were classified as low risk of bias. Clinical and imaging predictors including age, disease duration, preoperative mJOA scores, cervical imaging parameters, intramedullary hyperintense signals, comorbidities and smoking status were assessed following unified diagnostic and evaluation standards in both single-center and multicenter studies. We could not confirm universal outcome assessor blinding for most included studies, as only a minority of primary articles explicitly documented blinded evaluation of postoperative endpoints. For studies without clear reporting of blinding procedures, we did not automatically assume blinded assessment when scoring PROBAST, and this limitation was fully considered in our overall risk judgment. All predictors can be readily acquired in routine clinical practice, satisfying the data collection requirements for clinical application of prediction models.

Regarding the outcomes domain, only the study by Kadanka et al. (17) was rated as high risk of bias, and the remaining 11 studies were low risk of bias. Kadanka et al. (17) subjectively determined the progression from asymptomatic spinal cord compression to symptomatic DCM merely based on clinical symptoms and conventional imaging manifestations, without adopting internationally recognized standardized evaluation systems such as the mJOA score. Objective quantitative criteria were absent. In addition, the follow-up interval was inconsistent across individuals and loss to follow-up occurred in several cases, which easily led to outcome misclassification and subjective assessment bias.

By contrast, other studies defined outcomes using mJOA scores, JOA improvement rates or MCID at fixed postoperative time points, in accordance with widely accepted cut-off values. Uniform outcome criteria were applied to all participants, and assessors were blinded to baseline predictor information. The time interval between predictor measurement and outcome follow-up was rationally set with rigorous chronological logic, resulting in a standardized outcome evaluation system.

The analysis domain was rated as high risk of bias for all 12 studies. Five studies (5, 15, 16, 21, 22) satisfied the Events Per Variable (EPV ≥ 20) (25) standard proposed by methodological guidelines. The remaining six studies (10, 17–19, 23, 24) contained excessive candidate predictors relative to limited outcome events and thus failed to meet the EPV threshold, while Saggi et al. (20) did not report the total number of outcome events for EPV calculation.

In the analysis domain, improper handling of continuous variables was identified as a recurrent methodological flaw. Specifically, three studies (5, 16, 17) used cut-off-based dichotomization or categorization, and one study (22) applied restricted cubic splines without justification, all of which deviate from the optimal practice of retaining continuous variables in their original form. According to PROBAST signalling question 13, such practices may lead to loss of information and model misspecification, thereby contributing to the high risk of bias in this domain. Additionally, some studies developed models based on single-center cohorts or specific subgroups rather than all eligible participants, which further limits generalizability and introduces unmeasured confounding.

For missing data handling, significant heterogeneity and methodological deficiencies were observed across the included studies. Five studies (15, 20, 21, 23, 24) adopted complete case analysis, which may introduce selection bias and reduce effective sample sizes when missing data are not completely at random. Only one study (5) used multiple imputation, while four studies (10, 18, 19, 22) employed other explicit imputation or correction strategies (e.g., KNN imputation, negative value assignment, or canonical variate regression). Notably, two studies (16, 17) did not report any missing data handling strategy. The predominance of complete case analysis and the lack of justification for chosen imputation methods—particularly in studies with unreported missing proportions—constitute a major source of bias in the analysis domain, as such practices may distort the original data distribution and compromise model validity (PROBAST signalling question 15).

As detailed in the model performance section, although five studies (5, 16, 18, 21, 22) reported both discrimination and calibration, and four (5, 10, 23, 24) completed external validation, most models lacked rigorous calibration reporting and external testing. The predominant use of complete case analysis for missing data and the lack of justification for handling continuous variables further compounded analytical bias. Notably, one machine learning model (23) included 1,032 predictors with only 80 outcome events (EPV ≈ 0.08), indicating severe overfitting risk.

### TRIPOD reporting quality assessment

We quantitatively assessed the completeness of reporting for all 12 eligible prediction studies using the full 22-item TRIPOD checklist, with item-by-item scoring data provided in Supplementary Table S2. The overall TRIPOD compliance rate of the included literatures ranged from 59.1 to 81.8%. Three studies (5, 16, 22) achieved the highest reporting compliance of 81.8%, whereas three studies (10, 17, 24) exhibited the lowest compliance at 59.1%. Across all included articles, the most commonly underreported TRIPOD items were pre-specified sample size planning (Item 8), formal model calibration assessment (Item 14), independent external validation cohort construction (Item 16), and full raw regression/model coefficient tables (Item 22).

## Discussion

This systematic review provides a comprehensive overview and critical appraisal of existing prediction models for disease progression and postoperative outcomes in DCM. After systematic screening, 12 eligible studies were included, encompassing both prospective and retrospective cohort designs from four countries. Modeling approaches ranged from conventional logistic regression to diverse machine learning and ensemble algorithms, with discriminative performance (AUC) varying from 0.53 to 0.93. Despite the broad range of modeling techniques, several consistent findings emerged. Age, baseline neurological function assessed by mJOA/JOA scores, symptom duration, smoking status, and comorbidities were identified as the most consistent independent predictors across studies. These findings align with prior systematic reviews on DCM prognostic factors (5), which similarly underscored the prognostic dominance of these readily obtainable clinical variables. However, the emergence of radiomic features in two recent studies (23, 24) represents a novel direction; whether these high-dimensional imaging biomarkers provide meaningful incremental predictive value over conventional clinical and MRI parameters remains uncertain, as neither study conducted head-to-head comparisons with simpler reference models. This evidence gap warrants particular attention in future research.

A major challenge in synthesizing evidence across the included studies is the substantial heterogeneity that permeates nearly every aspect of study design and conduct. This heterogeneity originates from multiple, interrelated sources: divergent study designs (seven prospective vs. five retrospective cohorts); heterogeneous target populations (one study predicting preclinical progression in asymptomatic cord compression, versus 11 studies addressing postoperative recovery); variable outcome definitions (mJOA/JOA recovery, VAS improvement, MCID attainment, or composite trajectories); inconsistent follow-up durations (ranging from 3 months to 3 years); and diverse modeling algorithms (from simple logistic regression to complex ensemble methods). This multifaceted heterogeneity has two important implications. First, it precludes quantitative meta-analysis, restricting our synthesis to qualitative appraisal. Second, and more importantly, it cautions against direct cross-model comparisons, as a model’s apparent superiority in AUC may simply reflect differences in outcome definitions or case mix rather than genuine methodological advances. Future prediction modeling studies in DCM should adopt consensus-driven, standardized outcome definitions and follow-up protocols to facilitate meaningful comparisons across populations and settings.

Despite the proliferation of modeling approaches, the methodological quality of the included studies remains suboptimal. Notably, all 12 studies were rated as having high overall risk of bias under PROBAST, driven predominantly by deficiencies in the analysis domain. Among the most concerning deficiencies is the pervasive neglect of calibration assessment. Although AUC has been the de facto standard for evaluating discriminative performance, only five studies reported any form of calibration evaluation, whereas the remaining seven relied exclusively on AUC. This imbalance is problematic because AUC reflects only a model’s ability to rank-order patients by risk, not whether the predicted probabilities correspond to actual event rates—a property essential for clinical decision-making. Several factors may explain this widespread omission. First, many clinical investigators remain unfamiliar with calibration metrics or inadvertently equate AUC with comprehensive performance evaluation. Second, several included studies adopted a “proof-of-concept” approach prioritizing algorithmic novelty over methodological rigor, where calibration assessment was deemed secondary or dispensable. Third, in small-sample studies with EPV < 20—which constituted a substantial proportion of our included cohort—poor calibration is virtually guaranteed due to overfitting, and reporting calibration would expose this limitation. These explanations collectively underscore the need for enhanced methodological training and for journals to enforce stricter reporting requirements, particularly regarding calibration, as mandated by the TRIPOD statement.

The optimal choice between traditional regression and machine learning approaches remains an open question in DCM prediction modeling, yet our data permit several nuanced observations. Conventional logistic regression models, when built on a limited set of prespecified clinical predictors [e.g., the three-variable model by Tetreault et al. (5) comprising age, baseline mJOA, and symptom duration], achieved AUCs of 0.74–0.77 with rigorous internal and external validation. By contrast, machine learning models occasionally attained higher AUCs—up to 0.93 in Song et al. (21)—but this apparent superiority requires cautious interpretation. First, ML models typically incorporated far more predictors [e.g., 1,032 radiomic features in Zhang et al., (23)], raising legitimate concerns about overfitting, particularly given limited outcome events (EPV ≈ 0.08 in that study). Second, none of the ML studies conducted external validation beyond internal cross-validation, whereas the highest-performing clinical models were validated in independent external cohorts. Third, the performance advantage of complex ML models often shrinks dramatically upon external testing—a phenomenon well documented in prediction modeling literature but difficult to confirm here due to the paucity of external validation among ML studies. Fourth, ML models frequently sacrifice interpretability, a critical requirement for clinical decision-making, without offering proportionally greater predictive accuracy. Therefore, based on the current evidence, parsimonious clinical models with rigorous calibration and external validation appear more clinically deployable than high-dimensional radiomic ML models, which remain predominantly at the exploratory stage pending multi-center validation. The marginal discriminative gain of radiomic ML models (max AUC 0.93 vs. 0.77 of clinical regression) is offset by severe generalizability and implementation limitations, making the incremental clinical benefit negligible under routine hospital conditions.

Translating these prediction models into clinical practice faces substantial barriers that extend beyond statistical performance. First, radiomic-based models require T2*-weighted imaging sequences, specialized feature extraction software, and trained personnel—resources not universally available, particularly in primary and secondary hospitals or in resource-limited settings. Second, radiomic features exhibit limited inter-center reproducibility due to inherent variations in MRI hardware, acquisition protocols, and post-processing pipelines, raising concerns about external generalizability even if internal performance appears promising. Third, even for simpler clinical models, none of the included studies performed systematic assessment of clinical effectiveness or decision impact following model development, such as decision curve analysis or implementation studies. Without such evaluations, the real-world value of these models—whether they improve preoperative counseling, surgical decision-making, or patient outcomes—remains unknown. To address these translational gaps, future research should prioritize: (1) validation of existing simple clinical models [particularly Tetreault et al’s. (5) three-variable model] in geographically diverse external cohorts; (2) development of bedside-friendly nomograms or risk calculators that require no specialized software; and (3) prospective implementation studies that evaluate whether model-guided risk stratification actually improves clinical decision-making or patient-centered outcomes.

The strengths of this systematic review include its rigorous adherence to CHARMS, PROBAST, and TRIPOD guidelines; comprehensive searches across major English databases; independent dual-reviewer screening, data extraction, and bias assessment with cross-verification; and coverage of both single-center and multicenter cohorts. Unlike prior qualitative reviews that simply catalogued methodological defects, this work further provides stratified, actionable research recommendations differentiated by predictor accessibility, model calibration quality, and validation completeness, offering guidance to both clinical spine surgeons and computational investigators pursuing high-value follow-up investigations.

Several limitations of this review should also be acknowledged. First, inclusion was restricted to English-language publications; Chinese databases were not searched, potentially introducing language bias and missing relevant studies published in Chinese journals. Second, substantial heterogeneity in study design, outcome definitions, and modeling algorithms precluded quantitative meta-analysis, restricting our synthesis to a qualitative systematic review. Third, as a secondary analysis based on published summary data, individual patient-level raw data were unavailable; we could not perform independent re-modeling or external validation to verify the reported performance metrics, limiting our ability to assess true generalizability. Fourth, publication bias is likely, as studies with poor model performance may have been less likely to be published; the reported AUCs may therefore overestimate the true predictive ability of these models in routine clinical practice. Future meta-analyses or individual-participant-data (IPD) meta-analyses, when available, could address some of these limitations.

## Conclusion

This systematic review demonstrates that current DCM prediction models are characterized by substantial methodological heterogeneity and high overall risk of bias, predominantly driven by inadequate sample-event matching, inappropriate handling of continuous variables, and poor calibration reporting. Parsimonious clinical models based on age, baseline mJOA, and symptom duration currently offer the best balance of performance and interpretability, whereas radiomic machine learning models have not demonstrated sufficient added value to justify their complexity and implementation barriers. Future research should prioritize external validation of existing simple models and development of bedside-friendly tools, with strict adherence to TRIPOD and PROBAST reporting standards.

## Data Availability

The original contributions presented in the study are included in the article/Supplementary material, further inquiries can be directed to the corresponding authors.
