# Supplementary material for: Prediction models for progression and outcomes in degenerative cervical myelopathy: a systematic review and critical appraisal
Source: Front Neurol. 2026 Aug 21;17:1894454. doi: 10.3389/fneur.2026.1894454 (PMC13541547; doi:10.3389/fneur.2026.1894454)
Supplement: Supplementary file 1 [file Table_1.DOCX]

### Supplementary File S1

### Full search strategies

#### 1. PubMed

#1 ("Degenerative Cervical Myelopathy"[MeSH Terms] OR DCM OR cervical spondylotic myelopathy)

#2 (prediction model OR nomogram OR risk score OR machine learning OR radiomics OR predictive analytics)

#3 (disease progression OR postoperative prognosis OR surgical outcome OR functional recovery OR MCID)

#4 #1 AND #2 AND #3

Filters: English, from inception to May 1, 2026

#### 2. Embase

('degenerative cervical myelopathy' OR DCM OR cervical spondylotic myelopathy) AND ('prediction model' OR nomogram OR risk score OR machine learning OR radiomics) AND (progression OR postoperative outcome OR functional recovery OR MCID)

Limits: English, database inception – 01 May 2026

#### 3. Web of Science

TS=("degenerative cervical myelopathy" OR DCM OR "cervical spondylotic myelopathy") AND

TS=("prediction model" OR nomogram OR risk score OR machine learning OR radiomic) AND

TS=(progression OR postoperative prognosis OR surgical outcome OR MCID) Timespan: All years; Language: English
