# Supplementary material for: Prediction models for progression and outcomes in degenerative cervical myelopathy: a systematic review and critical appraisal
Source: Front Neurol. 2026 Aug 21;17:1894454. doi: 10.3389/fneur.2026.1894454 (PMC13541547; doi:10.3389/fneur.2026.1894454)
Supplement: Supplementary file 2 [file Table_2.DOCX]

**Supplementary Table S2.**

**Item-by-item TRIPOD 22-checklist reporting quality assessment of the included studies.**

| **First author (Year)** | **Item1** | **Item2** | **Item3** | **Item4** | **Item5** | **Item6** | **Item7** | **Item8** | **Item9** | **Item10** | **Item11** | **Item12** | **Item13** | **Item14** | **Item15** | **Item16** | **Item17** | **Item18** | **Item19** | **Item20** | **Item21** | **Item22** | **Overall compliance (%)** |
| --- | --- | --- | --- | --- | --- | --- | --- | --- | --- | --- | --- | --- | --- | --- | --- | --- | --- | --- | --- | --- | --- | --- | --- |
| Zhou 2025 | FR | FR | FR | FR | FR | FR | FR | NR | PR | FR | FR | PR | FR | NR | FR | FR | PR | NR | PR | FR | PR | NR | 59.1 |
| Kadanka 2017 | FR | FR | FR | FR | FR | FR | FR | FR | PR | FR | FR | NR | FR | NR | NR | NR | NR | PR | PR | FR | NR | FR | 59.1 |
| Jaja 2023 | FR | FR | FR | FR | FR | FR | FR | NR | PR | FR | FR | FR | FR | FR | FR | NR | FR | FR | PR | FR | FR | FR | 81.8 |
| Zhang 2022 | FR | FR | FR | FR | FR | FR | FR | NR | PR | FR | FR | PR | FR | NR | FR | FR | NR | NR | PR | FR | NR | NR | 59.1 |
| Merali 2019 | FR | FR | FR | FR | FR | FR | FR | NR | FR | FR | FR | FR | FR | NR | FR | NR | NR | NR | PR | FR | FR | NR | 68.2 |
| Khan 2021 | FR | FR | FR | FR | FR | FR | FR | NR | FR | FR | FR | FR | FR | FR | FR | NR | NR | NR | PR | FR | FR | NR | 72.7 |
| Song 2024 | FR | FR | FR | FR | FR | FR | FR | NR | FR | FR | FR | FR | FR | FR | FR | FR | NR | NR | PR | FR | FR | NR | 77.3 |
| Stephens 2023 | FR | FR | FR | FR | FR | FR | FR | NR | FR | FR | FR | FR | FR | FR | FR | NR | FR | PR | PR | FR | NR | FR | 81.8 |
| Zhang 2021 | FR | FR | FR | FR | FR | FR | FR | NR | FR | FR | FR | FR | FR | NR | FR | FR | NR | NR | PR | FR | FR | NR | 72.7 |
| Tetreault 2015 | FR | FR | FR | FR | FR | FR | FR | NR | FR | FR | FR | FR | FR | FR | FR | FR | FR | PR | PR | FR | NR | FR | 81.8 |
| Evaniew 2020 | FR | FR | FR | FR | FR | FR | FR | NR | NR | FR | FR | NR | FR | NR | NR | FR | NR | NR | PR | FR | FR | FR | 63.6 |
| Saggi 2025 | FR | FR | FR | FR | FR | FR | FR | NR | PR | FR | FR | FR | FR | NR | FR | FR | FR | NR | PR | FR | NR | NR | 68.2 |

**Notes:** FR = Fully reported; PR = Partially reported; NR = Not reported. The overall compliance percentage was calculated as the number of fully reported TRIPOD items divided by all 22 items multiplied by 100%. TRIPOD, Transparent Reporting of a multivariable prediction model for Individual Prognosis Or Diagnosis.
